# Radar-Based Gesture Recognition Using Adaptive Top-K Selection and Multi-Stream CNNs

**DOI:** 10.3390/s25206324

**Published:** 2025-10-13

**Authors:** Jiseop Park, Jaejin Jeong

**Affiliations:** Department of Electronic Engineering, Kumoh National Institute of Technology, Gumi-si 39177, Republic of Korea; flamma0202@gmail.com

**Keywords:** FMCW radar, radar signal preprocessing, deep learning, hand gesture recognition, human–computer interaction, human–machine interface

## Abstract

With the proliferation of the Internet of Things (IoT), gesture recognition has attracted attention as a core technology in human–computer interaction (HCI). In particular, mmWave frequency-modulated continuous-wave (FMCW) radar has emerged as an alternative to vision-based approaches due to its robustness to illumination changes and advantages in privacy. However, in real-world human–machine interface (HMI) environments, hand gestures are inevitably accompanied by torso- and arm-related reflections, which can also contain gesture-relevant variations. To effectively capture these variations without discarding them, we propose a preprocessing method called Adaptive Top-K Selection, which leverages vector entropy to summarize and preserve informative signals from both hand and body reflections. In addition, we present a Multi-Stream EfficientNetV2 architecture that jointly exploits temporal range and Doppler trajectories, together with radar-specific data augmentation and a training optimization strategy. In experiments on the publicly available FMCW gesture dataset released by the Karlsruhe Institute of Technology, the proposed method achieved an average accuracy of 99.5%. These results show that the proposed approach enables accurate and reliable gesture recognition even in realistic HMI environments with co-existing body reflections.

## 1. Introduction

The development of the Internet of Things (IoT) has driven innovation across domains such as smart homes, healthcare, transportation, industrial environments, and defense by seamlessly connecting people, objects, and the environment [1,2]. At the same time, the growing demand for hygienic and natural system control without physical contact has drawn increasing attention to contactless user interfaces [3,4,5]. In the field of human–computer interaction (HCI), technologies such as speech recognition and gesture recognition are being developed as alternatives to traditional interfaces, including keyboards, mouses, and touch screens [6]. However, speech recognition technologies remain vulnerable to voice variations and noisy environments, while also raising concerns about privacy [7,8]. In contrast, gesture recognition offers an intuitive form of user input, is free from language barriers, and can be effectively used in silent environments, thereby establishing itself as a core technology in HCI [9,10]. The advancement of deep learning architectures and optimization strategies has substantially improved gesture recognition performance across diverse modalities [11]. In particular, progress in vision-based gesture recognition demonstrates that modern neural models can effectively capture subtle temporal and spatial variations, enabling accurate discrimination of complex motion patterns [12].

Numerous studies that combine optical vision methods—including RGB cameras [13], depth cameras [14], LiDAR [15], and thermal infrared cameras [16]—with deep learning have demonstrated excellent performance. However, such approaches are vulnerable to illumination changes and occlusion, and they process video or audio data that can be directly interpreted by humans, thereby raising privacy concerns [6]. In contrast, radar measures only reflect electromagnetic waves, so the data do not directly resemble human sensory inputs [17]. Owing to the penetrability of radio waves, radar is also unaffected by lighting or shadows and is relatively advantageous in terms of privacy [18]. In particular, mmWave frequency-modulated continuous-wave (FMCW) radar, with its wide bandwidth and fine temporal and spectral resolution, can simultaneously capture subtle variations in range and Doppler, making it highly suitable for implementing short-range gesture interfaces such as smart home control and in-vehicle human–machine interfaces (HMIs).

Previous studies on FMCW radar-based gesture recognition have shown excellent performance by combining diverse signal processing pipelines with deep learning techniques [19,20,21,22,23,24,25,26]. However, many of these works relied on restricted environments and assumed the suppression of non-hand reflections, which differs from real HMI scenarios [18]. In practice, sensors are oriented toward the user, and hand gestures are accompanied by torso- and arm-related reflections. Gestures arise from a kinematic chain involving the shoulder, arm, and hand [27]. Therefore, torso-related signals may also contain meaningful variations functionally related to gestures. Motivated by this observation, we propose a method that effectively summarizes body-related components to preserve gesture information, thereby enabling reliable gesture recognition in realistic HMI conditions. The main contributions of this study are threefold: (i) an entropy-based Adaptive Top-K Selectionpreprocessing method to mitigate information loss and attenuation, (ii) a Multi-Stream EfficientNetV2 architecture for jointly learning range and Doppler trajectories, and (iii) radar-specific data augmentation with a training optimization strategy to maximize model performance. To the best of our knowledge, no prior work has systematically compared different 1D vector compression methods for Range–Doppler Images, and this study is the first to provide such a comparative analysis along with a novel adaptive algorithm. Through these contributions, the proposed method was experimentally validated using datasets, achieving stable and accurate gesture recognition even under realistic conditions where body reflections coexist, and demonstrating improved performance compared to existing methods. The remainder of this paper is organized as follows. Section 2 reviews related works and formulates the problem statement. Section 3 introduces the proposed methodology, including Range–Doppler Image generation, the entropy-based Adaptive Top-K Selection algorithm, and the Multi-Stream EfficientNetV2 architecture with radar-specific data augmentation. Section 4 describes the experimental setup, training procedure, and evaluation results. Finally, Section 5 concludes the paper and discusses future research directions.

## 2. Related Works and Problem Statement

### 2.1. Related Works

Radar-based gesture recognition techniques have been studied using various radar sensors, sensing methods, and algorithms. Tiwari et al. [28] utilized wearable UWB antennas to classify six arm gestures based on S-parameters and achieved a high accuracy of 99.76% using Extreme Gradient Boosting (XGB). Despite their excellent performance, wearable systems require direct attachment to the body, which reduces usability in practical applications.

As a result, fully contactless approaches have become mainstream, where radar sensors directly sense the user. A representative example is Google’s Soli project [29], which employed a 60 GHz mmWave FMCW radar with a Random Forest classifier to recognize gestures, followed by many subsequent studies that developed high-accuracy systems. Wang et al. [24] proposed an end-to-end model combining a 2D CNN and a Long Short-Term Memory (LSTM) network using per-frame Range–Doppler Maps (RDMs, used interchangeably with RDIs in this paper) as input, achieving 88% accuracy in subject-independent classification of 11 gestures. Choi et al. [25] extracted one-dimensional motion profiles from RDMs as LSTM inputs and reported a high accuracy of 98.48% for 10 gestures. Hayashi et al. [26] employed the second-generation Soli chip to develop RadarNet, a multi-head architecture combining 2D CNNs and LSTMs capable of simultaneously predicting left–right and up–down swipe gestures, achieving over 99% accuracy on pre-segmented datasets.

RNN-based models have also been widely adopted. Suh et al. [19] introduced the Projected RDM (PRDM) and used it as LSTM input for real-time recognition of seven gestures, reaching over 91% accuracy. These results demonstrate that sequential spatiotemporal information is vital for robust gesture recognition. Extending this idea, Zhang et al. [21] combined a 3D CNN with an LSTM using an FMCW radar, achieving 96% accuracy over eight gestures. However, the use of 3D CNNs significantly increases memory consumption and computational complexity.

Beyond RNN-based architectures, several studies have investigated the extraction of spatiotemporal feature maps from frame-wise radar data through dimensionality reduction and their use with 2D CNNs. Chmurski et al. [22] proposed a method to derive range–time, Doppler–time, and angle–time maps from FMCW radar signals and applied a 2D CNN for gesture classification, achieving a test accuracy of 98.13% across eight gestures. In a similar direction, Ahmed et al. [20] introduced a multi-stream 2D CNN for digit recognition that jointly processes range–time, Doppler–time, and angle–time maps, reporting 94.2% accuracy. However, generating angle–time maps requires direction-of-arrival (DOA) estimation via algorithms such as MUSIC or MVDR [30], which are computationally expensive due to eigen-decomposition and matrix inversion.

Although these studies have demonstrated high accuracy under controlled conditions, most datasets were collected in limited environments (e.g., confined boxes, simple backgrounds) and mainly included isolated hand gestures [19,20,22,25]. In realistic HMI scenarios, sensors face users in open spaces. With wider detection ranges, reflections from the torso and upper body inevitably coexist with hand gestures, resulting in self-reflections (DC leakage), static background signals, non-hand body responses, and electronic noise [31]. To suppress such noise, Moving Target Indicator (MTI) techniques such as moving average and high-pass filters have been widely applied [32,33,34]. However, these techniques may remove functionally important slow gesture components and subtle torso movements, leading to potential information loss. Moreover, conventional methods typically involve computationally expensive operations such as eigen-decomposition and matrix inversion. In contrast, our method preserves gesture-relevant information by summarizing body-related components rather than simply discarding them. Furthermore, it employs a multi-stream 2D CNN architecture that avoids eigen-decomposition and matrix inversion, enabling robust and reliable gesture recognition even in realistic HMI conditions.

### 2.2. Dataset and Problem Statement

The dataset used in this study was introduced by Antes et al. [35] and provides raw FMCW radar data corresponding to seven gesture types plus a No Gesture class [36]. Hereafter, this dataset is referred to as the KIT radar gesture dataset.

**Figure 1:**
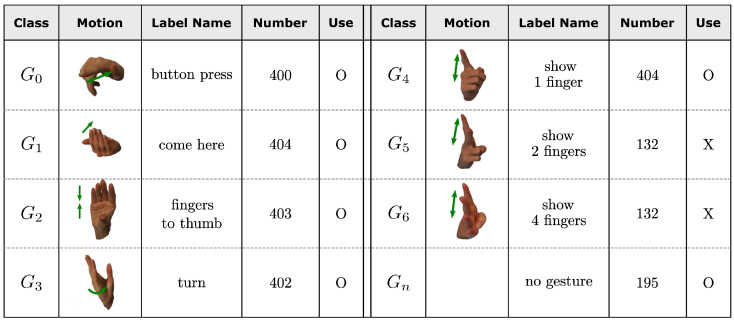
Dynamic hand gestures from the KIT radar gesture dataset [36]. In this study, six gestures marked with “O” in the “Use” column were selected for classification.

In the original dataset paper [35], Part 1 and Part 2 were combined to increase the number of samples per class. In addition, $G_{5}$ show 2 fingers, and $G_{6}$ show 4 fingers, were excluded, as these gestures are highly similar to $G_{4}$ show 1 finger, contain relatively fewer samples, and are more prone to misclassification in practical applications. Therefore, this study also followed the same configuration and used six gesture classes for classification. After excluding seven None Value Data samples, a total of 2208 samples were used for experiments. Figure 1 summarizes the detailed information of each gesture class.

**Figure 2:**
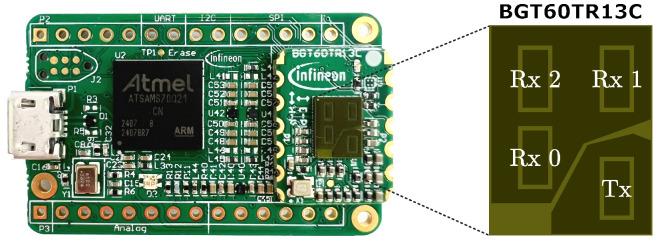
BGT60TR13C radar demo board with one transmitting (Tx) and three receiving (Rx) antennas [37].

Data acquisition was conducted using the BGT60TR13C demo board from Infineon Technologies AG, which is equipped with one transmit antenna and three receive antennas [37]. Figure 2 shows the radar demo board with the transmit and receive antennas highlighted.

The FMCW radar transmits chirp waveforms with linearly modulated frequencies, and the reflected signals exhibit a frequency shift relative to the transmitted signal due to the time delay. This frequency difference generates a beat frequency containing the target’s range information. The transmit antenna periodically emits chirp signals, while the three receive antennas capture the reflected signals. These received signals are mixed with the transmitted signal to produce intermediate frequency (IF) signals, which are then digitized by an analog-to-digital converter (ADC) and stored as raw FMCW radar data for each channel.

**Figure 3:**
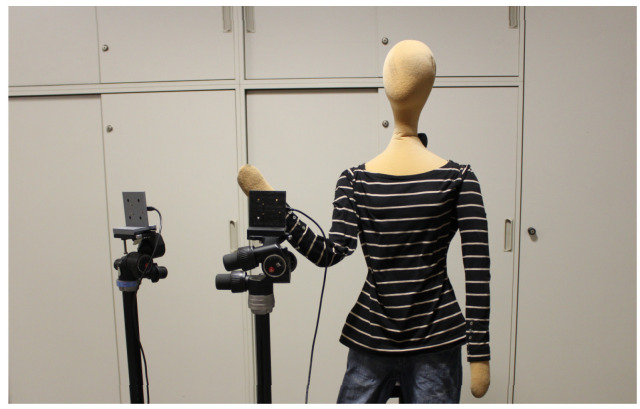
Measurement environment of the KIT radar gesture datase (Setup 3). This figure shows one representative configuration as an example (reproduced from [36]).

According to the original paper introducing the KIT radar gesture dataset, the subject’s torso is included within the radar sensing range, as illustrated in Figure 3, providing conditions similar to real HMI environments. In such a measurement setting, gesture-related information may exist not only in the hands and arms but also in the torso; therefore, removing torso-related reflections carries the risk of discarding useful signals associated with gestures.

In this context, the main challenge of this study is to propose a preprocessing method that effectively preserves gesture information in radar data containing both hand gestures and body-related reflections and to improve deep learning classification performance.

## 3. Proposed Methodology

### 3.1. Range–Doppler Image Generation

Each sample collected by the radar sensor (see Figure 2) consists of multiple frames, and each frame forms a 2D real-valued array with $N_{C}$ chirps and $N_{S}$ samples. A total of three receive channels are available, and identical data structures are collected for each channel. The signal of one frame from a specific receive channel a∈{0,1,2} can be expressed as follows:

(1) $$X_{\mathrm{raw}}^{\left(f,a\right)}=\left[\begin{array}{ccc} x_{1,1}^{\left(f,a\right)} & \ldots & x_{1,N_{S}}^{\left(f,a\right)}\\ \vdots & \ddots & \vdots \\ x_{N_{C},1}^{\left(f,a\right)} & \ldots & x_{N_{C},N_{S}}^{\left(f,a\right)} \end{array}\right]\in R^{N_{C}\times N_{S}}$$

where $x_{c,s}^{\left(f,a\right)}$ denotes the real-valued signal collected at the *s*-th sample of the *c*-th chirp in the *f*-th frame of receiving channel *a*. The sensor measurement parameters of the dataset are shown in Table 1, with each frame consisting of $N_{C}=600$ chirps and each chirp containing $N_{S}=128$ samples. These measurement parameters follow standard FMCW radar specifications, whose physical interpretations (e.g., the relation of $N_{S}$, $N_{C}$, and $T_{C}$ to range and Doppler resolutions) have been described in prior works [32,38]. Here, we summarize only the parameters directly used in our preprocessing pipeline. In the following description, the frame and channel indices are fixed, and the signal is simply denoted as $X_{\mathrm{raw}}\in R^{N_{C}\times N_{S}}$.

From $X_{\mathrm{raw}}$, in order to extract range and velocity components generated by multiple reflectors, a Range–Doppler Image (RDI) is generated across the three receiving channels. The RDI generation process is as follows. First, DC leakage and static clutter components are suppressed by applying 2D mean removal. $X_{\mathrm{raw}}$ is processed by subtracting the column-wise mean and then the row-wise mean, resulting in a zero-mean matrix $X_{\mathrm{zero}}$. Each chirp of $X_{\mathrm{zero}}$ is multiplied by a Hamming window to reduce sidelobes [39], and a 1D Fast Fourier Transform (FFT) is applied along the sample axis to extract range profiles. This 1D FFT applied along the sample axis is referred to as the Range FFT. To improve range resolution, a zero-padded FFT of $N_{\mathrm{FFT}}=448$ points is applied to the original $N_{S}=128$ samples. Finally, only the positive frequency components are retained, yielding a spectrum with $N_{R}=224$ range bins. The result of the Range FFT is given as

(2) $$\begin{array}{c} X_{\mathrm{range}}\left(c,r\right)=\sum_{s=1}^{N_{S}}w_{r}\left(s\right)\cdot X_{\mathrm{zero}}\left(c,s\right)\cdot \exp\left(-j\frac{2\pi \;r\cdot s}{N_{\mathrm{FFT}}}\right),\;r\in \left[1,\frac{N_{\mathrm{FFT}}}{2}\right] \end{array}$$

where $w_{r}$ denotes the Hamming window applied in the range direction. For each range bin *r*, a Hamming window $w_{d}$ is applied in the chirp direction, followed by a 1D FFT to extract Doppler components. This process is called the Doppler FFT. Since the FFT output is complex-valued, its magnitude is taken to obtain a matrix representing Doppler intensity at each range bin. The result of the Doppler FFT is expressed as

(3) $$\begin{array}{c} X_{\mathrm{doppler}}\left(d,r\right)=\left|\sum_{c=1}^{N_{C}}w_{d}\left(c\right)\cdot X_{\mathrm{range}}\left(c,r\right)\cdot \exp\left(-j\frac{2\pi \;d\cdot c}{N_{C}}\right)\right| \end{array}$$

**Figure 4:**
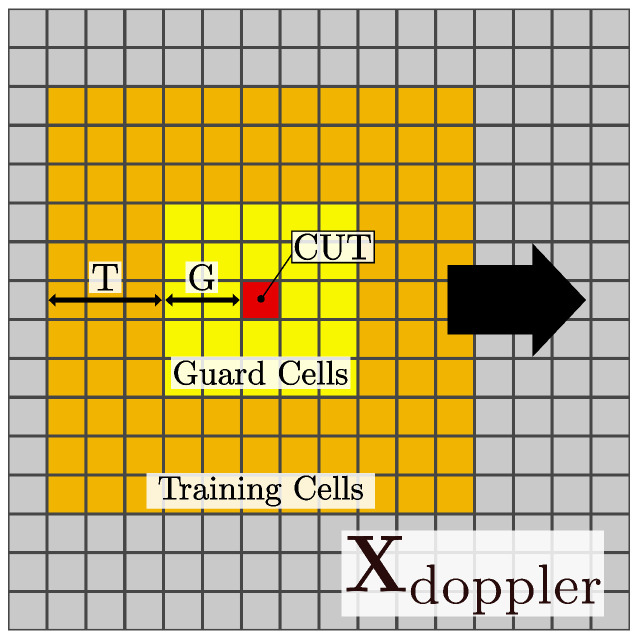
Window-based 2D CA-CFAR kernel example.

Subsequently, to emphasize target reflection signals while suppressing background noise and ghost targets, the Cell-Averaging Constant False Alarm Rate (CA-CFAR) algorithm is applied [40]. In this study, CA-CFAR is implemented on the Doppler spectrum $X_{\mathrm{doppler}}$ of the Range–Doppler plane by sliding a 2D window-based kernel. Figure 4 illustrates the core structure of the 2D CA-CFAR window configuration.

At this stage, for each Cell Under Test (CUT), a binary mask M(d,r) is generated by comparing the cell value with a dynamic threshold. The dynamic threshold is obtained by multiplying a scaling factor α with the local noise level $\mu_{\left(d,r\right)}$ estimated from surrounding reference cells.

(4) $$M\left(d,r\right)=\left\{\begin{array}{cc} 1, & \mathrm{if}\;X_{\mathrm{doppler}}\left(d,r\right)>\alpha \cdot \mu_{\left(d,r\right)}\\ 0, & \mathrm{otherwise} \end{array}\right.$$

where $\mu_{\left(d,r\right)}$ denotes the mean amplitude calculated over ${\left(2T+2G+1\right)}^{2}-{\left(2G+1\right)}^{2}$ training cells, excluding both the guard cells and the CUT. In this study, the CA-CFAR parameters were empirically set to T=20, G=2, and α=3.0. Then, the Doppler magnitude spectrum $X_{\mathrm{doppler}}$ is multiplied by the binary mask and transformed through logarithmic scaling to produce the final $\mathrm{RDI}\in R^{N_{D}\times N_{R}}$:

(5) $$\mathrm{RDI}=\log\left(1+X_{\mathrm{doppler}}⊙M\right)=\left[\begin{array}{ccc} \mathrm{RDI}(1,1) & \ldots & \mathrm{RDI}(1,N_{R})\\ \vdots & \ddots & \vdots \\ \mathrm{RDI}(N_{D},1) & \ldots & \mathrm{RDI}(N_{D},N_{R}) \end{array}\right]\in R^{N_{D}\times N_{R}}$$

where ⊙ represents the Hadamard (element-wise) product between two matrices of the same dimension. The term log(1+·) avoids undefined values when the input is zero and prevents low-amplitude components from being completely suppressed during logarithmic scaling.

### 3.2. Adaptive Top-K Selection Based RTM and DTM Generation

The RDI is a 2D array of size $N_{D}\times N_{R}$ that represents the energy distribution of gestures and background reflections within a single frame. A data sample from one channel has a 3D structure in which RDIs are arranged sequentially across frames. Each frame-level RDI is compressed into a 1D vector along either the Doppler (row) or Range (column) dimension, and these vectors are stacked along the temporal axis to generate a 2D time-series map. These time-series maps are defined as the Doppler-Time Map (DTM) and the Range-Time Map (RTM).

#### 3.2.1. Compression Methods from RDI to the 1D Vector

**Figure 5:**
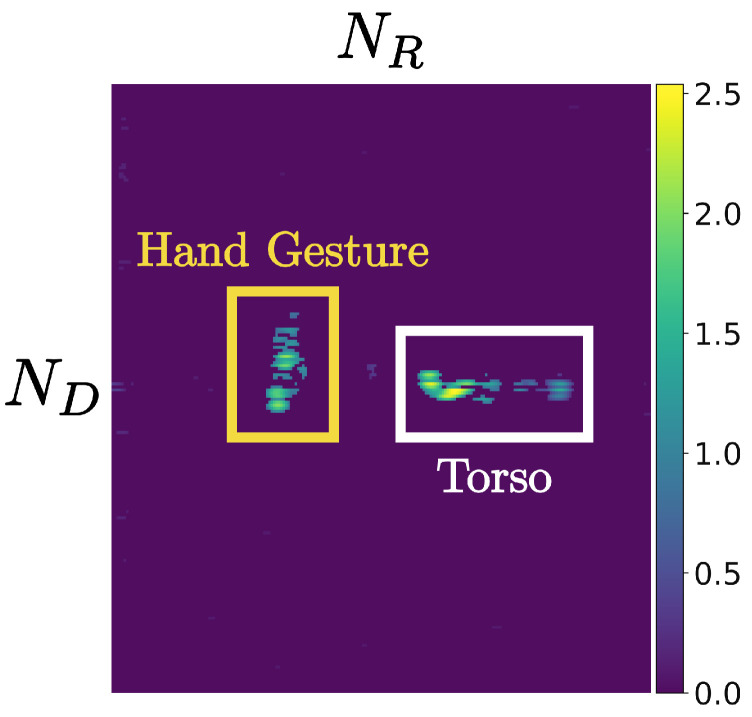
Example of a Range–Doppler Image (RDI) without removing body-related reflections. Multiple peak clusters are observed, corresponding to reflections from both the hand gesture and the torso. The highlighted regions indicate the localized energy distributions of gesture and torso components.

Since motion-induced components from non-hand body parts were not removed in the preprocessing step described in Section 3.1, the RDI exhibits multiple peak clusters, as shown in Figure 5. In this example, the cluster on the left corresponds to reflections from the hand gesture, while the cluster on the right originates from the torso. The highlighted boxes indicate localized energy distributions, with the yellow box marking gesture-related peaks and the white box marking torso-related reflections.

Several methods can be used to compress a frame-level RDI into a 1D vector:

(a) Summation across rows or columns:
  This method utilizes all available information and is robust to noise. However, strong peaks may be diluted by background energy, leading to blurred features.
(b) Maximum extraction from each row or column: This approach emphasizes the strongest peaks, resulting in clearer features but may incorrectly highlight noise peaks or body-related reflections.
(c) Slicing at the maximum peak position: This method suffers from severe information loss, and if the maximum peak originates from noise rather than the gesture, feature distortion may occur.
(d) Top-*K* summation after sorting by magnitude: This method preserves information around strong reflectors and mitigates noise effects. However, the balance between information preservation and noise suppression depends on the choice of *K*.

**Figure 6:**
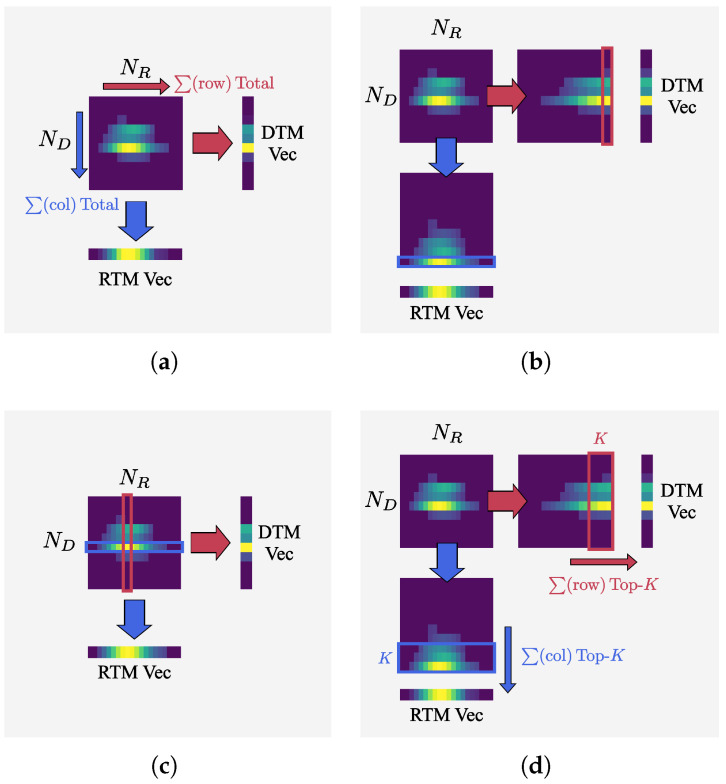
Illustration of four compression methods for reducing RDIs into 1D vectors: (**a**) summation across rows or columns; (**b**) maximum extraction from each row or column; (**c**) slicing at the maximum peak position; (**d**) Top-*K* summation after sorting by magnitude.

Figure 6 shows four compression methods using a reduced RDI for illustration. Arrows indicate the compression direction: horizontal for Doppler–time (DTM) and vertical for range–time (RTM). Figure 6a–d present the resulting 1D vectors obtained by each method. Considering their respective advantages and disadvantages, this study adopts method Figure 6d, the Top-*K* summation approach, to compress RDIs into 1D vectors.

#### 3.2.2. Adaptive Top-K Selection Algorithm

Using a fixed *K* value to sum only the Top-*K* components cannot reflect the diverse energy dispersion characteristics of each row or column vector in an RDI. To address this issue, this study proposes the Adaptive Top-K Selection algorithm, which quantifies the dispersion of each vector and automatically selects an optimal *K*.

CA-CFAR masking converts the RDI into a sparse matrix in which most values are zero, while valid reflections remain only around peak clusters (Figure 5). In such sparse peak clusters, the most informative pixels are typically surrounded by sidelobe components, and the fewer surrounding nonzero pixels there are, the less informative the corresponding bin tends to be. Therefore, an appropriate dispersion measure is required to quantify the distribution of values in each row or column vector. For this purpose, Shannon entropy [41] was adopted, defined for a normalized vector $p_{i}=v_{i}/\left(\sum_{j}v_{j}+\epsilon \right)$ as

$$\begin{array}{c} H=-\sum_{i=1}^{n}p_{i}\log p_{i}. \end{array}$$

This entropy ranges from H=0 when all energy is concentrated in a single bin (perfectly peaked vector) to H=logn when it is uniformly distributed across all bins (maximally diffuse vector).

As *H* decreases, the vector is more likely dominated by sidelobe-like components of lower importance, whereas a higher *H* indicates a vector with more evenly distributed and potentially meaningful components. Accordingly, the *K*-selection strategy is defined as

- Peak distribution (low entropy): A larger *K* is selected to include surrounding components, thereby diluting sidelobe peaks and enhancing the relative emphasis on vectors containing richer information.
- Diffuse distribution (high entropy): A smaller *K* is chosen to avoid the inclusion of unnecessary zeros, preserving only the core components and maintaining structural clarity.

**Figure 7:**
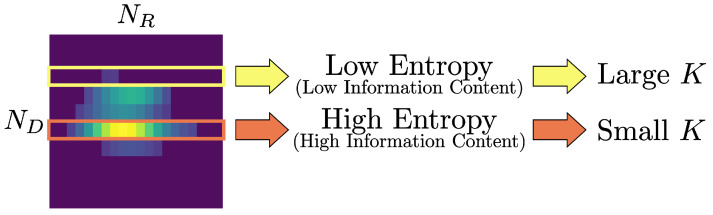
Example of entropy-based *K* determination for row vectors in a reduced RDI sample.

Figure 7 illustrates an example of entropy-based *K* determination, and Table 2 summarizes the step-by-step procedure. Low-entropy vectors use a larger *K* to include adjacent entries, whereas high-entropy vectors use a smaller *K* to retain only core components.

This rule implies that, in Top-*K* summation, a larger *K* includes more zero or near-zero elements. As a result, the normalized output becomes visually blurred. In contrast, a smaller *K* produces a sharper result by concentrating on the strongest components. Thus, the entropy-based selection adaptively balances attenuation and emphasis according to the information content of each vector. For RDIs containing multiple peak clusters, even when entropy is high and a small *K* is chosen, $K_{\min}$ should be set greater than 1 to reduce information loss. Conversely, if entropy is low and *K* is large, setting $K_{\max}$ to be excessively high may also cause attenuation. In this study, we heuristically set $K_{\min}=5$ and $K_{\max}=20$.

Once *K* is determined, the Top-*K* elements of each column (range bin) are summed to form an RTM vector, and the Top-*K* elements of each row (Doppler bin) are summed to form a DTM vector. This entire procedure—entropy-based *K* selection followed by summation—is hereafter referred to as the Adaptive Top-K Summation.

#### 3.2.3. RTM and DTM Generation

**Figure 8:**
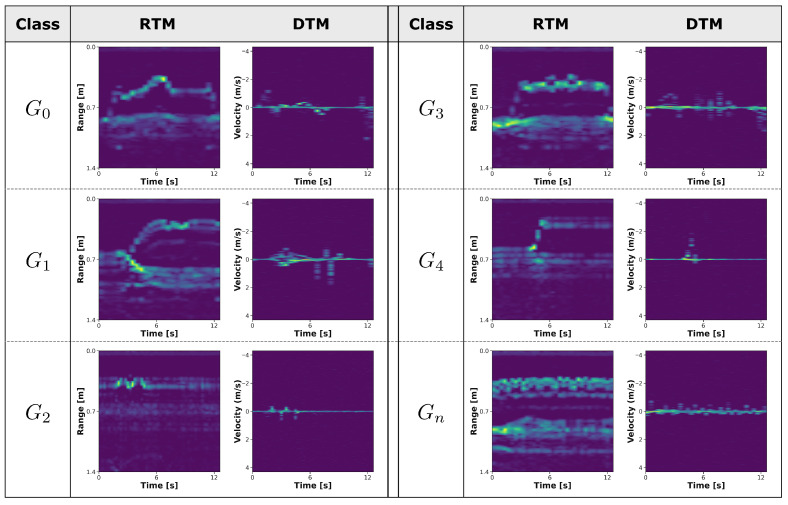
Examples of RTM and DTM generated from Rx0 for each gesture class.

Stacking the RTM and DTM vectors from all frames in chronological order yields two distinct images that represent the temporal range trajectory and velocity trajectory of the gesture. After applying Min-Max Normalization to each image and resizing them to 224×224, the final RTM and DTM corresponding to a single receiving channel are obtained. Figure 8 shows examples of RTM and DTM generated from Rx0 for each gesture class.

### 3.3. Multi-Stream EfficientNetV2

For each receiving channel, one RTM and one DTM are generated, yielding three RTMs and three DTMs. The three RTMs are stacked into a single three-channel image for 2D CNN input, and the same is carried out for the DTMs. Thus, each sample is represented by two three-channel images, which are independently processed by 2D CNN backbones and then fused for final classification.

**Figure 9:**
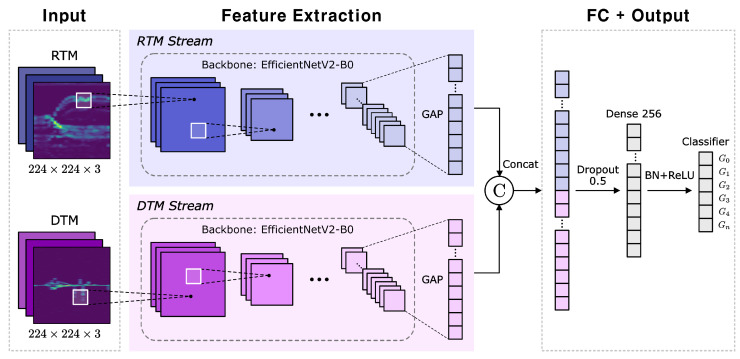
Framework of the proposed Multi-EffNetV2.

In this study, the EfficientNetV2-B0 was adopted as the backbone for each RTM and DTM stream, as it provides a good balance between accuracy and efficiency. EfficientNet, EfficientNetV2, and their variants are convolutional neural networks (CNNs) that have been widely applied to diverse computer vision classification tasks across various domains [42,43,44,45,46]. EfficientNetV2 builds upon its predecessor, the EfficientNet architecture [47], and provides high accuracy, strong parameter efficiency, and fast GPU training speed [48]. Pre-trained weights from the ImageNet dataset were not used; instead, only the architecture was employed. The model was implemented using TensorFlow and Keras libraries and initialized through the tf.keras.applications.EfficientNetV2B0 function. Figure 9 illustrates the architecture of the proposed Multi-EffNetV2 network. The RTM and DTM streams each take an input image of size 224×224×3, extract features using EfficientNetV2-B0, and then perform final classification into six gesture classes through fully connected layers. The proposed Multi-EffNetV2 contains a total of 12.38 million trainable parameters and requires approximately 2.89 GFLOPs per inference. Although heavier than lightweight backbones such as MobileNet, the model is still much smaller than vision transformers or 3D CNNs, offering a practical balance between accuracy and efficiency.

### 3.4. Radar-Specific Data Augmentation

The RTM and DTM used as inputs to the proposed model are two-dimensional time-series images (spectrograms) obtained from the FMCW radar domain. Therefore, directly applying conventional geometric augmentation techniques commonly used in image classification (e.g., rotation, flipping, color transformation) may distort the inherent meaning embedded in the time–frequency structure of the original signals, potentially leading to degraded performance. To address this issue, inspired by the time- and Doppler-domain scaling concepts proposed by Kern et al. [49], we propose three radar-specific augmentation methods:

1. RTM Vertical Shift: The RTM is randomly shifted along the range axis (vertical direction), with the shift magnitude defined as $\gamma_{r}∼U\left(-30,+30\right)$ pixels. Empty regions created by the shift are filled with zeros, and the same shift is applied to all three receiving channels. This simulates changes in the absolute distance between the sensor and the user.
  $$\begin{array}{c} {\mathrm{RTM}}^{\prime }\left(r,t\right)=\mathrm{RTM}\left(r+\gamma_{r},t\right) \end{array}$$
2. RTM and DTM Horizontal Stretch: The RTM and DTM are scaled along the time axis (horizontal direction) using a random scaling factor $\gamma_{t}∼U\left(0.7,1.3\right)$. The transformation is centered, and empty regions are zero-padded. The same scaling is applied to all six RTM/DTM images. This simulates variations in gesture repetition cycles and overall motion speed.
  $$\begin{array}{c} {\mathrm{RTM}}^{\prime }\left(r,t\right)=\mathrm{RTM}\left(r,\gamma_{t}\cdot t\right),\;{\mathrm{DTM}}^{\prime }\left(d,t\right)=\mathrm{DTM}\left(d,\gamma_{t}\cdot t\right) \end{array}$$
3. DTM Vertical Stretch: The DTM is scaled along the Doppler axis (vertical direction) using a random scaling factor $\gamma_{d}∼U\left(0.7,1.3\right)$. The transformation is centered, and empty regions are zero-padded. The same scaling is applied to all three receiving channels. This reflects instantaneous variations in gesture speed.
  $$\begin{array}{c} {\mathrm{DTM}}^{\prime }\left(d,t\right)=\mathrm{DTM}\left(\gamma_{d}\cdot d,t\right) \end{array}$$

**Figure 10:**
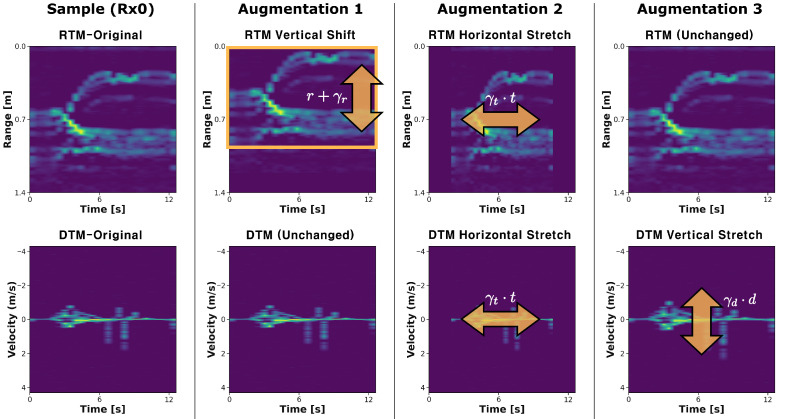
Examples of the proposed radar-specific data augmentation methods applied to RTM and DTM from Rx0 of a single sample. The first column shows the original RTM and DTM, while the subsequent columns illustrate (1) RTM vertical shift, (2) RTM/DTM horizontal stretch, and (3) DTM vertical stretch. Each augmentation simulates variations in range, motion cycle, or gesture speed, thereby enhancing the diversity of the training data.

Examples of the three augmentation methods are shown in Figure 10, illustrated using the RTM and DTM generated from Rx0 of a single sample. Each augmentation is applied to samples at the batch level during training and randomly re-sampled at each epoch, contributing to improved generalization performance of the model.

## 4. Experiments

### 4.1. Experimental Setup

This section describes the experimental environment used for training and evaluating the proposed Multi-EffNetV2 model. All experiments were conducted on a PC equipped with an Intel Core i9-13900KF CPU (24 cores, 32 threads), 128 GB DDR5 RAM, an NVIDIA GeForce RTX 4090 GPU (24 GB VRAM), and Ubuntu 20.04.6 LTS (WSL2). The software environment consisted of Python 3.10, TensorFlow 2.13, CUDA 11.8, and cuDNN 8.6. The dataset, comprising 2208 samples, was divided into training and testing sets at an 8:2 ratio using a stratified split, and evaluation was performed exclusively on the test set. To mitigate class imbalance, class weights were applied during training. All experiments were conducted with the same hyperparameter settings: the AdamW optimizer ($\lambda =1\times 10^{-4}$) [50], an initial learning rate of $1\times 10^{-3}$, a batch size of 32, and a total of 70 epochs. To ensure statistical reliability, model training and evaluation were repeated five times with different random seeds for each preprocessed dataset.

### 4.2. Training and Evaluation

**Figure 11:**
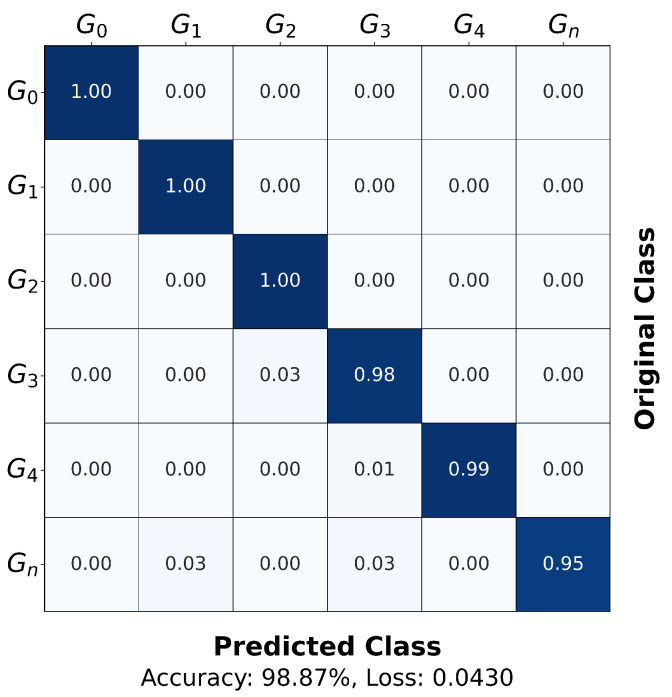
Normalized confusion matrix of the test set for the proposed Adaptive Top-K Summation with the Multi-EffNetV2 model, corresponding to the run with the highest accuracy among five repeated experiments.

This section evaluates the performance of the Adaptive Top-K Selection algorithm. To eliminate performance gains attributable to data augmentation or training optimizations, no additional strategies such as augmentation methods or learning rate schedulers were applied. Figure 11 shows the normalized confusion matrix of the test set obtained from the Multi-EffNetV2 model trained with the dataset preprocessed using Adaptive Top-K Summation, corresponding to the run that achieved the highest accuracy among five repeated experiments. The proposed method demonstrated excellent classification performance across all gesture classes, achieving the highest accuracy of 98.87% in the best run.

The evaluation accuracy was compared among datasets preprocessed using the methods in Figure 6 and those preprocessed with the proposed Adaptive Top-K Summation. Table 3 summarizes the highest, lowest, and average accuracies.

Conventional compression methods have clear drawbacks: method (a) dilutes salient peaks with background energy, while methods (b) and (c) risk information loss by retaining only local maxima. Method (d) achieved its best result at K=10 with an average accuracy of 97.96%, but it remained limited by applying the same *K* uniformly to all vectors. In practice, finding such an empirically optimal *K* requires exhaustive experimentation and does not generalize well across different datasets or signal conditions. In contrast, the proposed Adaptive Top-K dynamically adjusts *K* using the Shannon entropy of each vector, preserving meaningful components within peak clusters, handling multi-peak distributions more robustly and ultimately achieving the best performance with an average accuracy of 98.60% and a highest accuracy of 98.87%.

### 4.3. Performance Optimization

**Figure 12:**
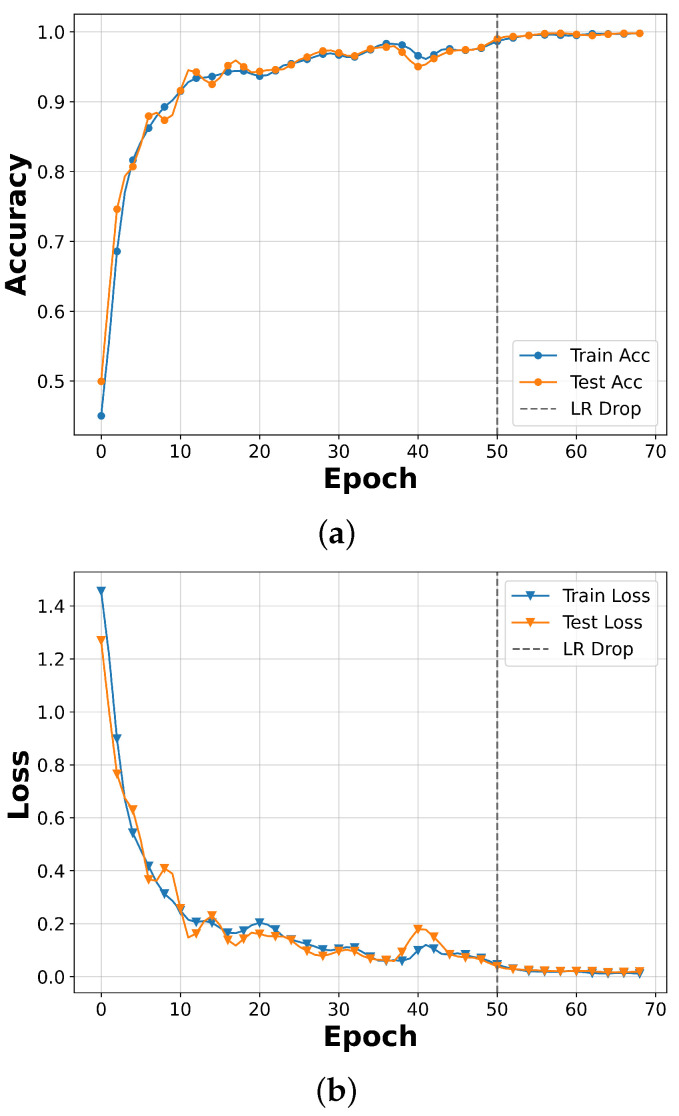
Training curves of the Multi-EffNetV2 model with Radar-Specific Data Augmentation and learning rate scheduler applied. Results correspond to the best-performing run among five repetitions: (**a**) accuracy curve and (**b**) loss curve. The learning rate was reduced by a factor of 10 at epoch 50, leading to stabilized convergence and accuracy exceeding 99%.

**Figure 13:**
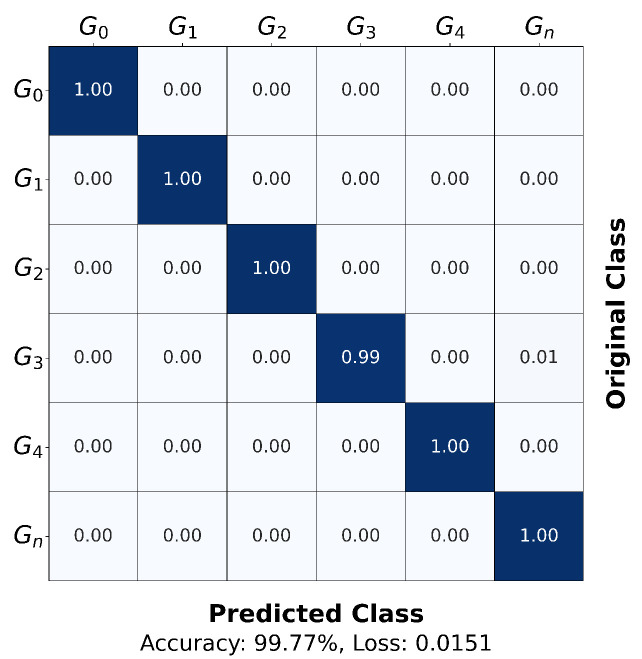
Normalized confusion matrix for the test set obtained from the best-performing run among five repetitions with Adaptive Top-K Summation, Radar-Specific Data Augmentation, and learning rate scheduler applied. The proposed configuration achieved the highest evaluation accuracy of 99.77%.

In this section, we enhance the final performance of the Multi-EffNetV2 model by applying radar-specific data augmentation (Section 3.4) and a learning rate scheduler to the dataset preprocessed with Adaptive Top-K Summation. The scheduler reduced the learning rate by a factor of 10 at epoch 50, improving accuracy and facilitating loss convergence in the later training stage. Figure 12 illustrates the training curves of the best-performing run among five repetitions, showing that the model converged rapidly in the early epochs and stabilized after the learning rate decay. Figure 13 presents the normalized confusion matrix of the same run, which achieved the highest overall evaluation accuracy of 99.77%, demonstrating consistently high classification performance across all gesture classes, with notable improvements in the low-sample $G_{n}$ class.

Across the five repeated experiments, the proposed configuration achieved the highest evaluation accuracy of 99.77%, the lowest of 99.10%, and an average of 99.50%. From the best run, the macro-average accuracy across all gesture classes was 99.83%. Compared to the baseline reported in the dataset paper, where ResNet trained on a reduced subset (G_0_–G_4_, G_n_) with Short-Time Fourier Transform (STFT) preprocessing achieved per-class accuracies ranging from 71.4% to 96.0% (Table 4 [35]), the proposed Multi-EffNetV2 substantially outperformed prior results. This highlights the effectiveness of the proposed preprocessing pipeline and demonstrates that, when combined with an optimized deep learning architecture, it can fully exploit the potential of the dataset.

## 5. Discussion and Conclusions

This study aimed to improve classification performance in FMCW radar environments by preserving gesture information despite coexistence with torso- and arm-related reflections. To address this challenge, the study proposed (i) an entropy-based Adaptive Top-K Selection algorithm to mitigate information loss and attenuation, (ii) a Multi-Stream EfficientNetV2 architecture to jointly exploit range and Doppler trajectories, and (iii) radar-specific data augmentation with a training optimization strategy to further enhance model performance. The proposed method, evaluated on the KIT radar gesture dataset, achieved the highest evaluation accuracy of 99.77% in the best run, with an average accuracy of 99.50% across five repeated experiments. From the best-performing run, the macro-average accuracy across gesture classes reached 99.83%, demonstrating its high effectiveness and notable improvements in the low-sample $G_{n}$ class.

Nevertheless, this study has several limitations. First, the entropy-based preprocessing requires additional computation for every frame in order to determine the adaptive *K* value, which increases the preprocessing overhead. Second, while the proposed Multi-Stream EfficientNetV2 achieved strong performance, it contains 12.38 million trainable parameters and requires 2.89 GFLOPs per inference, which is compact compared to state-of-the-art vision transformers or 3D CNNs but still higher than lightweight models typically deployed on resource-constrained embedded hardware. These factors indicate that further research is needed to design more efficient pipelines that reduce preprocessing cost and model complexity, thereby enabling seamless deployment on edge devices.

Moreover, the present work focused on single-user, isolated gesture recognition in general environments. Extending the approach to multi-user and multi-gesture scenarios, as well as handling continuous gesture streams, remains an open challenge. Future work will explore preprocessing algorithm acceleration together with compression strategies such as model lightweight design and quantization to minimize latency and memory usage. We also plan to investigate real-time interactive applications where gesture recognition is integrated into broader HMI systems. These directions will not only address efficiency and deployment concerns but also expand the applicability of radar-based gesture recognition to practical, real-world use cases.

## Figures and Tables

**Table 1:** Radar measurement parameters of the BGT60TR13C sensor used in the KIT radar gesture dataset [36].

Parameter	Value
Frequency band $f_{\min}∼f_{\max}$	57.5–64.5 GHz
Sampling rate $f_{S}$	1 MHz
Chirp repetition time $T_{C}$	300 μs
Number of samples $N_{S}$	128
Number of chirps $N_{C}$	600
Range resolution ΔR	2.1 cm
Maximum unambiguous range $R_{\max}$	1.4 m
Velocity resolution Δv	1.5 cm/s
Maximum unambiguous velocity $v_{\max}$	4.3 m/s

**Table 2:** Adaptive Top-K Selection Algorithm.

Step	Description
Input	Vector $v\in R^{n}$, $K_{\min}$, $K_{\max}$
Output	Adaptive *K* value
Parameter	ϵ: a small positive constant to prevent division by zero
(1)	Compute $p_{i}=\frac{v_{i}}{\sum v_{i}+\epsilon }$ for i=1,…,n.
(2)	Compute entropy: $H=-\sum_{i=1}^{n}p_{i}\log p_{i}$.
(3)	Normalize entropy: $H_{\mathrm{norm}}=\frac{H}{\log n}$.
(4)	Compute $K=\lfloor \left(1-H_{\mathrm{norm}}\right)\left(K_{\max}-K_{\min}\right)+K_{\min}\rfloor$.
	end

**Table 3:** Comparison of evaluation accuracy among different compression methods over five repeated experiments. Highest, lowest, and average accuracies are reported: (**a**) summation across rows or columns; (**b**) maximum extraction from each row or column; (**c**) slicing at the maximum peak position; (**d**) Top-*K* summation with fixed values of K∈{5,10,15,20,25}; (**e**) proposed Adaptive Top-K Summation.

Evaluation Accuracy [%]	Compression Methods								
	Total Sum	Maximum Extraction	Peak Slicing	Top-5 Sum	Top-10 Sum	Top-15 Sum	Top-20 Sum	Top-25 Sum	Adaptive Top-K Sum
	(a)	(b)	(c)			(d)			(e)
Highest	96.38	97.06	92.99	97.74	98.19	97.74	97.51	96.61	98.87
Lowest	95.70	96.38	90.27	96.83	97.74	96.15	96.15	95.70	98.19
Average	96.06	96.65	91.36	97.29	97.96	96.97	96.79	96.20	98.60

## Data Availability

The radar gesture dataset used in this study, “A Flexible Data Set for Radar-based Gesture Recognition with an FC-FMCW Radar,” is publicly available at https://doi.org/10.35097/hCNNxXlJBdFTSYse. No new data were created in this study.

## Abbreviations

The following abbreviations are used in this manuscript:

1D FFT	One-Dimensional Fast Fourier Transform
2D CNN	Two-Dimensional Convolutional Neural Network
3D CNN	Three-Dimensional Convolutional Neural Network
ADC	Analog-to-Digital Converter
BN	Batch Normalization
CA-CFAR	Cell Averaging Constant False Alarm Rate
CUT	Cell Under Test
DOA	Direction-of-Arrival
DTM	Doppler-Time Map
GFLOPs	Giga Floating-Point Operations
FMCW	Frequency-Modulated Continuous-Wave
HCI	Human–Computer Interaction
HMI	Human–Machine Interface
IF	Intermediate Frequency
IoT	Internet of Things
LSTM	Long Short-Term Memory
MTI	Moving Target Indicator
MUSIC	Multiple Signal Classification
MVDR	Minimum Variance Distortionless Response
RDI	Range–Doppler Image
ReLU	Rectified Linear Unit
RGB	Red, Green, Blue
RNN	Recurrent Neural Network
RTM	Range-Time Map
STFT	Short-Time Fourier Transform
UWB	Ultra-Wideband
XGB	Extreme Gradient Boosting
